# Medicare Eligibility and Racial and Ethnic Disparities in Operative Fixation for Distal Radius Fracture

**DOI:** 10.1001/jamanetworkopen.2023.49621

**Published:** 2023-12-28

**Authors:** Trista M. Benítez, Zhongzhe Ouyang, Alexander N. Khouri, Joseph N. Fahmy, Lu Wang, Kevin C. Chung

**Affiliations:** 1 University of Michigan Medical School, Ann Arbor; 2 Section of Plastic Surgery, Department of Surgery, University of Michigan Medical School, Ann Arbor; 3 Department of Biostatistics, University of Michigan School of Public Health, Ann Arbor

## Abstract

**Importance:**

Medicare provides near-universal health insurance to US residents aged 65 years or older. How eligibility for Medicare coverage affects racial and ethnic disparities in operative management after orthopedic trauma is poorly understood.

**Objective:**

To assess the association of Medicare eligibility with racial and ethnic disparities in open reduction and internal fixation (ORIF) after distal radius fracture (DRF).

**Design, Setting, and Participants:**

This retrospective cohort study with a regression discontinuity design obtained data from the Healthcare Cost and Utilization Project all-payer statewide databases for Florida, Maryland, and New York. These databases contain encounter-level data and unique patient identifiers for longitudinal follow-up across emergency departments, outpatient surgical centers, and hospitals. The cohort included patients aged 57 to 72 years who sustained DRFs between January 1, 2016, and November 30, 2019. Data analysis was performed between March 1 and October 15, 2023.

**Exposure:**

Eligibility for Medicare coverage at age 65 years.

**Main Outcomes and Measures:**

Type of management for DRF (closed treatment, external fixation, percutaneous pinning, and ORIF). Time to surgery was ascertained in patients undergoing ORIF. Multivariable logistic regression and regression discontinuity design were used to compare racial and ethnic disparities in patients who underwent ORIF before or after age 65 years.

**Results:**

A total of 26 874 patients with DRF were included (mean [SD] age, 64.6 [4.6] years; 22 359 were females [83.2%]). Of these patients, 2805 were Hispanic or Latino (10.4%; hereafter, *Hispanic*), 1492 were non-Hispanic Black (5.6%; hereafter, *Black*), and 20 548 were non-Hispanic White (76.5%; hereafter, *White*) and 2029 (7.6%) were individuals of other races and ethnicities (including Asian or Pacific Islander, Native American, and other races). Overall, 32.6% of patients received ORIF but significantly lower use was observed in Black (20.2% vs 35.4%; *P* < .001) and Hispanic (25.8% vs 35.4%; *P* < .001) patients compared with White individuals. After adjusting for potential confounders, multivariable logistic regression analysis confirmed the disparity in ORIF use in Black (odds ratio [OR], 0.60; 95% CI, 0.50-0.72) and Hispanic patients (OR, 0.82; 95% CI, 0.72-0.94) compared with White patients. No significant difference in ORIF use was found among racial and ethnic groups at age 65 years. The expected disparity in ORIF use between White and Black patients at age 65 years without Medicare coverage was 12.6 percentage points; however, the actual disparity was 22.0 percentage points, 9.4 percentage points (95% CI, 0.3-18.4 percentage points) greater than expected, a 75% increase (*P* = .04). In the absence of Medicare coverage, the expected disparity in ORIF use between White and Hispanic patients was 8.3 percentage points, and this result persisted without significant change in the presence of Medicare coverage.

**Conclusions and Relevance:**

Results of this study showed that surgical management for DRF was popular in adults aged 57 to 72 years, but there was lower ORIF use in racial or ethnic minority patients. Medicare eligibility at age 65 years did not attenuate race and ethnicity–based disparities in surgical management of DRFs.

## Key Points

**Question:**

Is Medicare eligibility associated with reductions in racial and ethnic disparities in operative fixation after distal radius fracture (DRF)?

**Findings:**

In this cohort study of 26 874 patients who received treatment for DRF, eligibility for Medicare at age 65 years was not associated with a significant reduction in racial or ethnic disparities in operative management after DRF. Black and Hispanic patients had significantly reduced odds of surgery compared with White patients before and after age 65 years.

**Meaning:**

Findings of this study suggest that despite less confounding from insurance after age 65 years, there remains substantially lower use of surgical management for DRF in racial or ethnic minority patients.

## Teaser

This cohort study investigates whether age-related Medicare eligibility is associated with racial and ethnic disparities in the decision to use surgical management for distal radius fracture.

## Introduction

Distal radius fractures (DRFs) are common in adults aged 55 years or older and impose substantial morbidity.^1,2,3,4^ Operative management via open reduction and internal fixation (ORIF) has become increasingly popular in this age group because it is associated with earlier return to work and activities of daily living, thereby aligning with enhanced patient autonomy.^5,6^ However, research suggests social determinants of health play a role in surgical access and outcomes after orthopedic trauma.^7,8,9,10^

Although patients from racial and ethnic minority groups are less likely to undergo operative fixation and experience greater delays in care after trauma,^11^ research has found that less prominent disparities exist among Medicare beneficiaries.^12,13^ Consequently, previous studies have posited that there is an association of insurance coverage with race and ethnicity–based disparities in surgical care.^14,15,16^ Under this premise, it is unknown whether near-universal health care afforded through Medicare is associated with more equitable and timely access to surgical care after DRF.

Many database studies have investigated trends in DRF management and patient-level factors associated with internal fixation.^5,10,11,12,17^ Because these investigations commonly used commercial insurance databases that lacked race and ethnicity data,^5^ inpatient samples,^11^ or Medicare claims,^4,12,18^ there is limited insight into the potential disparities in ambulatory surgery for DRF and whether aging into Medicare mitigates these inequities. Using the Healthcare Cost and Utilization Project (HCUP) all-payer databases, this study aimed to assess the association of Medicare eligibility with race and ethnicity–based disparities in ORIF use after DRF. We applied a regression discontinuity design (RDD) using the age eligibility for Medicare to identify a discontinuity in surgical rates among White patients and racial or ethnic minority patients. Guided by a prior RDD study on health care access and disparities at age 65 years,^19^ we hypothesized that disparities in ORIF access exist but are attenuated at age 65 years when most US residents become eligible for Medicare.

## Methods

### Study Design and Data Source

This retrospective cohort study used the 2016 to 2019 State Emergency Department Database, State Ambulatory Surgery and Services Databases, and State Inpatient Databases for Florida, Maryland, and New York. State databases were linked to the data source and followed the study cohort. These HCUP all-payer databases contain encounter-level data and unique patient identifiers for longitudinal follow-up across different settings.^20^ Florida, Maryland, and New York are highly populated, diverse states that we selected based on data completeness and linkage variables.^21,22^ The institutional review board at the University of Michigan deemed this study exempt from review and the informed consent requirement because the data used were deidentified. We followed the Strengthening the Reporting of Observational Studies in Epidemiology (STROBE) reporting guideline.^23^

### Age-Based Discontinuity in Medicare Eligibility and Coverage Transitions 

Medicare is a federally financed program that provides near-universal health insurance for most US residents at age 65 years or older. Individuals become eligible at age 65 years if they or their spouse has worked and paid Medicare taxes for at least 10 years.^24^ As most individuals qualify for Medicare coverage under this criterion, a large age-based discontinuity in Medicare eligibility is created at age 65 years. Several studies have leveraged this discontinuity to estimate causal associations in Medicare coverage.^19,25,26,27^

### Study Cohort and Relevant Covariates

Adult patients with an emergency department (ED) primary diagnosis of closed unilateral DRF were identified using *International Statistical Classification of Diseases and Related Health Problems, Tenth Revision* (*ICD-10*) diagnostic codes, which were the same as those in previous studies of DRF management (eTable 1 in Supplement 1).^5,18,28,29^ To capture patients with isolated DRF, we excluded patients with concomitant injury and used *ICD-10* diagnostic codes (eTable 2 in Supplement 1).^30^ The study cohort included patients aged 57 to 72 years at time of ED visit between January 1, 2016, and November 30, 2019. This age range was chosen to identify DRF management patterns before and after age 65 years, consistent with prior RDD investigations of changes in health care use at time of Medicare eligibility.^25,31,32^

Patients with missing data on age, race and ethnicity, insurance type, or linkage variables were excluded. To isolate the outcome of Medicare eligibility at age 65 years, individuals who obtained Medicare coverage before reaching age 65 years were excluded from the primary analysis.^27^ We excluded state nonresidents to ensure we captured longitudinal care in state databases.^33^ Additionally, we excluded patients without DRF management within 14 days of injury. We used this end point to eliminate prior or subsequent fractures separate from the index encounter.^5^ At Michigan Medicine, most patients undergo management (surgical or other treatment) within 9 days of fracture, and 14 days has been used as an end point in prior investigations.^5^

Patient-level variables included age, race and ethnicity, sex, insurance type, urbanization of location, median household-income quartile, medical comorbidities represented by the Elixhauser Comorbidity Index score (range: 0 to >2, with the highest score indicating greater medical comorbidity),^34^ state of residence, and year of encounter. Race and ethnicity were obtained from the HCUP databases and were categorized as Hispanic or Latino of any race (hereafter, *Hispanic*), non-Hispanic Black (hereafter, *Black*), non-Hispanic White (hereafter, *White*), and Other (including Asian or Pacific Islander, Native American, and other races). White individuals accounted for 92.4% of the study sample, whereas Asian or Pacific Islander, Native American, and other individuals made up less than 8.0% of the sample and thus were classified as other and excluded from primary analysis because of small sample size.^19^

### Outcome Measures

The primary outcome was DRF management type. Patients were stratified and classified into 1 of 4 management groups by increasing invasiveness: closed treatment, external fixation, percutaneous pinning, and ORIF.^5^ For instance, if a patient received a cast in the ED but underwent ORIF several days later, the patient was classified into the ORIF group.^5,29^ At Michigan Medicine and in prior investigation settings, most patients who ultimately underwent ORIF first received closed treatment (eg, splint) at initial encounter.^5,29^ We used *Current Procedural Terminology* and *ICD-10* procedure codes to classify management (eTable 3 in Supplement 1).^30,35^ To assess differences in time to surgery, we calculated the number of days from initial presentation to date of definitive management.

### Statistical Analysis

Wilcoxon rank sum and Pearson χ^2^ tests were performed to identify differences in patient characteristics. To assess management type, we compared ORIF with all other modalities (closed treatment, external fixation and percutaneous pinning), consistent with prior studies.^5,18^ After stratifying by age groups, we assessed with χ^2^ tests the differences in ORIF use by race and ethnicity. We used multivariable logistic regression to estimate the odds of ORIF vs all other treatments by race and ethnicity. White race was the reference group against which to evaluate differences from Black and Hispanic races separately. Regression models controlled for age, sex, insurance type, median household-income quartile, urbanization of patient location, Elixhauser Comorbidity Index score, state of residence, and year of injury. We used unpaired, 2-tailed *t* tests to assess differences in time to ORIF by race and ethnicity.

In the RDD, we estimated the local average treatment effects of Medicare eligibility age by management type after DRF. We estimated the adjusted discontinuity at age 65 years using local linear regression with a uniform kernel, which allowed for modeling of different age groups below (57-64 years) and above (65-72 years) the discontinuity threshold. The bandwidth was selected using the Imbens and Kalyanaraman^36^ method. To overcome the issue of underestimating the SE of local average treatment effects due to discrete running variable, we clustered by the running variable and obtained bias-adjusted CIs.^37^ We documented how the estimates differed by Black, Hispanic, and White patients. Differences in adjusted discontinuities between racial and ethnic groups were calculated at age 65 years. The delta method was used to recover bias-adjusted CIs around that quantity.^19^

In a sensitivity analysis, we used fuzzy design to include noncompliers, defined as those who obtained Medicare coverage before age 65 years and those who did not apply for coverage after age 65 years. Given potential differences between traditional Medicare and Medicare Advantage, we performed an additional sensitivity analysis that excluded patients with Medicare Advantage.

Two-sided *P* < .05 indicated statistical significance. Data analysis was performed between March 1 and October 15, 2023, using R, version 4.3.0 (R Core Team).

## Results

**Figure 1.**
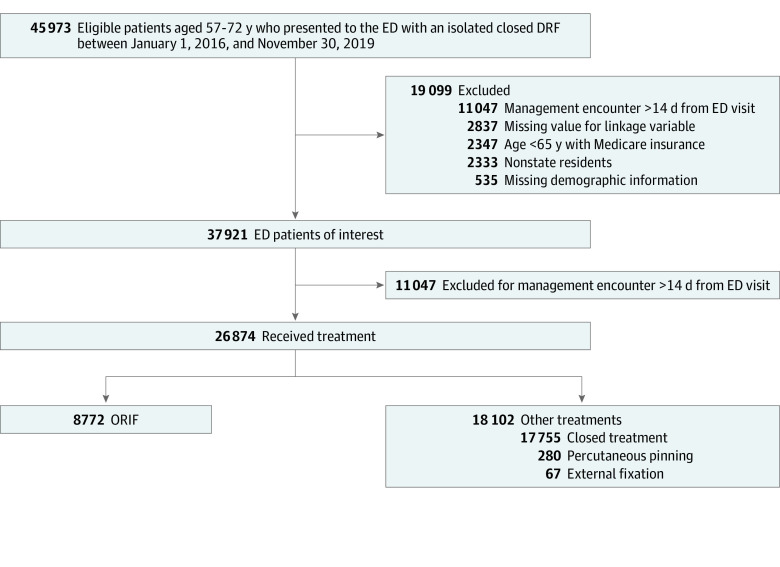
Study Cohort Flowchart DRF indicates distal radius fracture; ED, emergency department; and ORIF, open reduction and internal fixation.

The study cohort included 26 874 patients who received treatment (Figure 1). These patients had a mean (SD) age of 64.6 (4.6) years and comprised 22 359 females (83.2%) and 4515 males (16.8%), of whom 1492 (5.6%) were of Black, 2805 (10.4%) were of Hispanic, 20 548 (76.5%) were of White, and 2029 (7.6%) were of other race and ethnicity (Table 1). In total, 12 837 patients (47.8%) were aged 57 to 64 years and 14 037 (52.2%) were aged 65 to 72 years. Baseline characteristics of the study cohort stratified by race and ethnicity are presented in Table 1. Overall, 32.6% of patients underwent ORIF, with a similar proportion undergoing this management modality among patients in the 57 to 64 years and 65 to 72 years age groups. Significantly lower use was observed in Black (20.2% vs 35.4%; *P* < .001) and Hispanic (25.8% vs 35.4%; *P* < .001) patients compared with White individuals.

**Table 1.** Baseline Characteristics of the Study Cohort

Characteristic	Patients, No. (%)^a^				
	Overall (n = 26 874)	Black (n = 1492)	Hispanic (n = 2805)	White (n = 20 548)	Other (n = 2029)^b^
Age, mean (SD), y	64.6 (4.6)	63.7 (4.5)	64.3 (4.7)	64.8 (4.6)	64.1 (4.5)
Age category, y					
57-64	12 837 (47.8)	827 (55.4)	1411 (50.3)	9524 (46.4)	1075 (53.0)
65-72	14 037 (52.2)	665 (44.6)	1394 (49.7)	11 024 (53.6)	954 (47.0)
Sex					
Female	22 359 (83.2)	1128 (75.6)	2275 (81.1)	17 320 (84.3)	1636 (80.6)
Male	4515 (16.8)	364 (24.4)	530 (18.9)	3228 (15.7)	393 (19.4)
Insurance type					
Private	9919 (36.9)	508 (34.0)	849 (30.3)	9919 (48.3)	769 (37.9)
Traditional Medicare	6434 (23.9)	213 (14.3)	353 (12.6)	6434 (31.3)	351 (17.3)
Medicare Advantage	4784 (17.8)	243 (16.3)	693 (24.7)	4784 (23.3)	277 (13.7)
Medicaid	2234 (8.3)	276 (18.5)	391 (13.9)	2234 (10.9)	354 (17.4)
Self-pay	1604 (6.0)	146 (9.8)	281 (10.0)	1604 (7.8)	139 (6.9)
No charge	225 (0.8)	11 (0.7)	62 (2.2)	225 (1.1)	≤10^c^
Other^d^	1674 (6.2)	95 (6.4)	176 (6.3)	1674 (8.1)	≤10^c^
Medicare-Medicaid dual eligibility					
Yes	452 (16.8)	42 (2.8)	97 (3.5)	195 (1.0)	118 (5.8)
No	2703 (10.1)	115 (7.7)	150 (5.3)	2198 (10.7)	240 (11.8)
Unknown	23 719 (73.1)	1335 (89.5)	2558 (91.2)	18 155 (88.4)	1671 (82.4)
Median household-income national quartile					
0-25th percentile	6167 (22.9)	616 (41.3)	959 (34.2)	4180 (20.3)	412 (20.3)
26th-50th percentile	7609 (28.3)	322 (21.6)	784 (28.0)	6032 (29.4)	471 (23.2)
51st-75th percentile	6688 (24.9)	309 (20.7)	618 (22.0)	5251 (25.6)	510 (25.1)
76th-100th percentile	6410 (23.9)	245 (16.4)	444 (15.8)	5085 (24.7)	636 (31.3)
Urbanization of patient location					
Not metropolitan or micropolitan	596 (2.2)	16 (1.1)	≤10^c^	596 (2.9)	16 (0.8)
Micropolitan area	1114 (4.1)	14 (0.9)	≤10^c^	1114 (5.4)	65 (3.2)
Small metropolitan area	7895 (29.4)	228 (15.3)	292 (10.4)	7895 (38.4)	206 (10.2)
Large metropolitan area	17 269 (64.3)	1234 (82.7)	2476 (88.3)	17 269 (84.0)	1742 (85.9)
Elixhauser Comorbidity Index score					
0-1	22 888 (85.2)	1133 (75.9)	2325 (82.9)	17 676 (86.0)	1754 (86.4)
2	2818 (10.5)	261 (17.5)	355 (12.7)	2005 (9.8)	197 (9.7)
>2	1168 (4.3)	98 (6.6)	125 (4.5)	867 (4.2)	78 (3.8)
State of residence					
Florida	14 344 (53.4)	689 (46.2)	1786 (63.7)	11 462 (55.8)	407 (20.1)
Maryland	1932 (7.2)	257 (17.2)	76 (2.7)	1471 (7.2)	128 (6.3)
New York	10 598 (39.4)	546 (36.6)	943 (33.6)	7615 (37.1)	1494 (73.6)
Management type					
ORIF	8772 (32.6)	301 (20.2)	725 (25.8)	7278 (35.4)	468 (23.1)
All other treatments^e^	18 102 (67.4)	1191 (79.8)	2080 (74.2)	13 270 (64.6)	1561 (76.9)

Abbreviations: ED, emergency department; ORIF, open reduction and internal fixation.

^a^ Percentages may not total to 100 due to rounding.

^b^ Other included Asian or Pacific Islander, Native American, and other races.

^c^ Cell size counts of 10 or less are suppressed.

^d^ Other insurance types included Worker's Compensation; TRICARE; US Department of Veterans Affairs; other state and local government; Title V; and federal, state, and local Department of Corrections.

^e^ Treatments provided within 14 days of ED presentation and include closed treatment, external fixation, and percutaneous pinning.

Differences in management stratified by race and ethnicity and age range are depicted in Table 2. Overall, a greater proportion of White patients underwent ORIF compared with Black (34.7% vs 21.0%; *P* < .001) and Hispanic (34.7% vs 26.6%; *P* < .001) patients aged 57 to 64 years. After adjusting for potential confounders, the multivariable logistic regression results confirmed the disparity in ORIF rates in Black (odds ratio [OR], 0.60; 95% CI, 0.50-0.72) and Hispanic patients (OR, 0.82; 95% CI, 0.72-0.94) vs White patients. Among patients who received ORIF within 14 days of injury, the mean (SD) time to surgery was similar between White and Black patients (6.5 [3.9] days vs 6.6 [4.2] days; *P* = .12). Hispanic patients had reduced mean (SD) time to surgery compared with White individuals (5.9 [4.5] days vs 6.5 [3.9] days; *P* < .001). In those aged 65 to 72 years, we observed similar patterns, and the regression results confirmed the disparity between White and Black individuals and between White and Hispanic individuals (Table 2). Of those who received ORIF, Black patients had a longer mean (SD) time to surgery than White patients (7.4 [4.1] days vs 6.6 [3.7] days; *P* < .001). Hispanic patients had reduced mean (SD) time to surgery compared with White individuals (5.5 [4.4] vs 6.6 [3.7] days; *P* < .001).

**Table 2.** Management Differences Below and Above the Medicare Eligibility Age Threshold by Race and Ethnicity

	White patients	Black patients	*P* value^a^	Hispanic patients	*P* value^a^
**Age 57-64 y**					
ORIF vs all other treatments, No. (%)^b^	3309 (34.7)	174 (21.0)	<.001	375 (26.6)	<.001
Adjusted odds of ORIF vs all other treatments, OR (95% CI)^b,c^	1.00 [Reference]	0.60 (0.50-0.72)	<.001	0.82 (0.72-0.94)	.004
Time to ORIF within 14 d of ED presentation, mean (SD), d	6.5 (3.9)	6.6 (4.2)	.12	5.9 (4.5)	<.001
**Age 65-72 y**					
ORIF vs all other treatments, No. (%)^b^	3969 (36.0)	127 (19.1)	<.001	350 (25.1)	<.001
Adjusted odds of ORIF vs all other treatments, OR (95% CI)^b^	1.00 [Reference]	0.43 (0.31-0.60)	<.001	0.72 (0.58-0.91)	.005
Time to ORIF within 14 d of ED presentation, mean (SD), y	6.6 (3.7)	7.4 (4.1)	<.001	5.5 (4.4)	<.001

Abbreviations: ED, emergency department; OR, odds ratio; ORIF, open reduction and internal fixation.

^a^ Difference between White and Black patients and between White and Hispanic patients.

^b^ Treatments provided within 14 days of ED presentation and include closed treatment, external fixation, and percutaneous pinning.

^c^ Multivariable logistic regression model adjusted for age, sex, insurance, median household income, urbanization of patient location, Elixhauser Comorbidity Index score, state of residence, and year of injury.

**Figure 2.**
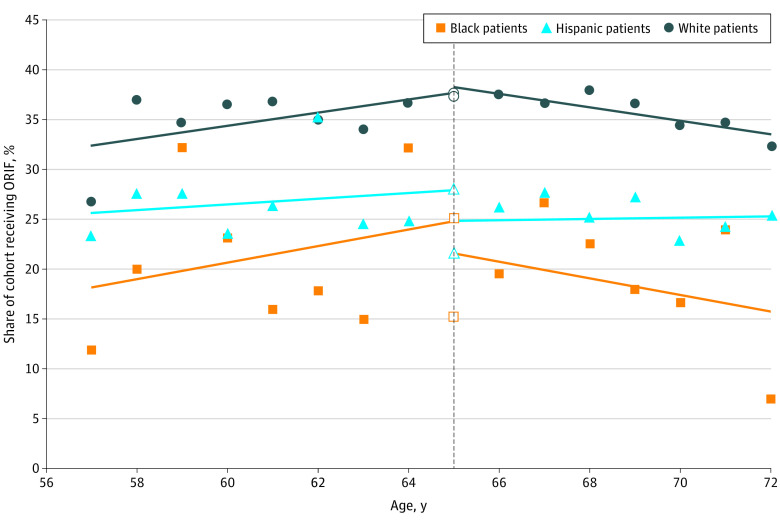
Rate of Open Reduction and Internal Fixation (ORIF) vs All Other Treatments Within 14 Days of Distal Radius Fracture by Race and Ethnicity For illustrative purposes, the local linear line of best fit based on optimal bandwidth selected for the regression discontinuity model is plotted separately for Black, Hispanic, and White patients. The black vertical dashed line represents the Medicare eligibility age threshold of 65 years.

The results of regression discontinuity analysis are presented in Table 3 and Figure 2. Overall, we observed no significant difference in ORIF use among racial and ethnic groups at age 65 years. The expected disparity between White and Black patients at age 65 years in the absence of Medicare coverage was 12.6 percentage points. However, the actual disparity was 22.0 percentage points, 9.4 percentage points (95% CI, 0.3-18.4 percentage points) greater than expected, a 75% increase (*P* = .04). In the absence of Medicare coverage, the expected disparity in ORIF use between White and Hispanic patients was 8.3 percentage points. With Medicare coverage, there was no significant change in the adjusted discontinuity in disparity, indicating a persistence in the disparity between White and Hispanic individuals at age 65 years. Results of sensitivity analyses are provided in the eAppendix and eTables 4 and 5 in Supplement 1.

**Table 3.** Medicare Eligibility Age–Related Discontinuities in Distal Radius Fracture Management by Race and Ethnicity

Racial or ethnic group	Expected mean (SD), %^a^	Adjusted discontinuity (95% CI), percentage points	Expected disparity, %^b^	Adjusted discontinuity in disparity (95% CI), percentage points	*P* value
Black	25.1 (4.32)	−7.7 (−16.2 to 0.7)	12.6	9.4 (0.3 to 18.4)	.04
Hispanic	28.0 (3.16)	−2.9 (−9.1 to 3.3)	9.7	4.5 (−2.5 to 11.5)	.21
White	37.7 (1.64)	1.6 (−1.6 to 4.9)	NA	NA	NA

Abbreviation: NA, not applicable.

^a^ The expected mean was age 65 years, based on local linear association between age and outcome (use of open reduction and internal fixation vs all other treatments). The expected mean contained the counterfactual outcome at age 65 years in the absence of treatment (eg, the expected outcome at age 65 years without Medicare).

^b^ The expected disparity was age 65 years, based on the local linear association between age and outcome (use of open reduction and internal fixation vs all other treatments). The expected disparity subtracted the expected mean for racially and ethnically minoritized groups from the expected mean for White populations at age 65 years.

## Discussion

The American Academy of Orthopedic Surgeons Clinical Practice Guidelines provide surgeons with evidence-based direction to deliver high-quality care that aligns with the Institute of Medicine’s 6 domains of health care quality: safe, effective, efficient, timely, patient-centered, and equitable.^38^ Equitable care is management that does not vary based on patient characteristics, including race and ethnicity.^38^ Yet, in this study, we found that Black and Hispanic individuals aged 57 to 72 years were less likely to undergo operative management after DRF compared with their White counterparts. This disparity persisted even after the age of Medicare eligibility (65 years) was reached, when there was less confounding from insurance and cost-sharing provisions.^19,39^ Instead, the findings suggest that other factors, such as structural barriers, patient preferences, and implicit bias, play a role in disparities in surgical management after this common orthopedic injury. Despite other studies reporting that Medicare eligibility was associated with narrowed racial and ethnic disparities in health care access and use,^19,26,40^ the regression discontinuity study that we performed demonstrated no greater parity in surgical management for DRF among patients of different races and ethnicities at age 65 years.

To our knowledge, only 2 prior studies have assessed the association of race and ethnicity with management outcomes for DRF and reported conflicting findings.^12,17^ Using Medicare claims data from 1998 to 2004, Fanuele et al^12^ found no difference in ORIF use between White and racial and ethnic minority Medicare beneficiaries. Because volar locking plates were relatively new at the time of their study, Fanuele et al^12^ observed a low internal fixation rate of 6%. Thus, racial and ethnic differences in management type may not have been as pronounced. Using a more contemporary sample, Chung et al^17^ found more prevalent ORIF use in Medicare patients, yet Black beneficiaries had 26% lower odds of surgery compared with White beneficiaries. Chung et al^17^ posited that the observed differences may reflect the lower prevalence of osteoporosis and subsequent risk for fracture collapse in Black vs White individuals,^17,41^ thus hypothesizing that surgeons could be reluctant to offer surgery to Black patients if a satisfactory outcome from casting was expected.^17^ However, epidemiologic factors cannot fully explain these differences. The prevalence of osteoporosis in Hispanic and White individuals is similar,^41,42,43^ yet we found in the present study that Hispanic patients were less likely to undergo surgery than White patients.

There is a myriad of factors that may explain the racial and ethnic disparities in surgical management for DRF in this study. First, even with less confounding from insurance differences, racial and ethnic minority patients may experience structural disparities in navigating outpatient surgical facilities and orthopedic care. Most patients who ultimately undergo ORIF for isolated DRF do so on an outpatient basis, and prior studies^21,44^ have found that ambulatory surgery centers are less accessible to racially and ethnically minoritized groups. It is also plausible that Black and Hispanic patients receive care in facilities with less robust referral pathways to orthopedic surgeons.^45^ Second, differences could reflect varying patient preferences. This factor has been well documented in total joint arthroplasty, wherein Black and Hispanic patients have underused this procedure compared with White patients despite a similar prevalence rate of hip and knee osteoarthritis.^46^ For instance, Suarez-Almazor et al^47^ found that White patients were 3 times more likely than Black patients and 6 times more likely than Hispanic patients to consider undergoing total knee arthroplasty if recommended by their physician. Although management for DRF differs from management for osteoarthritis in that it is nonelective, racial or ethnic minority patients may prefer nonoperative management because of underlying differences in perceived benefit, lack of awareness or understanding of surgical fixation, and potential mistrust.^47^ Third, physician-level factors, such as implicit bias, could lessen the offering of ORIF to racial or ethnic minority patients.^48^ Increasing attention to inequities in orthopedic surgical care coincides with a greater recognition of the low awareness of health disparities and lack of diversity within this surgical subfield.^49,50,51,52^ For instance, Adelani and O’Connor^52^ found that only 12% of surveyed orthopedic surgeons believed patients receive different health care because of race and ethnicity. Many have attributed these prevailing beliefs to the racial and ethnic underrepresentation within orthopedic surgery.^49,50,51,52^ In 2018, 84.7% of orthopedic surgeons in the US identified as White individuals, whereas only 1.9% and 2.2% identified as Black and Hispanic individuals, respectively.^49^ In the past decade, there were, on average, 4 Black and Hispanic hand surgery fellows each year, representing approximately 2% of total fellows, with a flat growth rate over this time.^53,54^ While health inequities are complex, racial or ethnic minority patients may more readily engage with physicians who share a similar background, upbringing, or cultural identity.^49,54,55^ Thus, enhancing diversity in these subfields, such as through participation in pipeline or summer immersion programs,^53^ could play a role in more equitable patient outcomes.

Although other studies have established differences in DRF treatment by socioeconomic status, the present study uniquely contributes to the literature by using an RDD to ascertain whether entry to Medicare was associated with greater parity in intensive management.^10,11,12,56,57^ The findings differed from other studies that used this quasi-experimental strategy to investigate Medicare eligibility and health equity. For instance, Wallace et al^19^ observed reduced disparities in primary care access and self-reported health between White and Black or Hispanic individuals at age 65 years. Similarly, Poulson et al^40^ reported that Black patients experienced a substantial decline in advanced stage at presentation after obtaining Medicare eligibility, thereby narrowing the disparities gap between Black and White patients with colon cancer. Findings from the present study most likely differed from these studies for several reasons. First, past investigations focused on routine care processes typically rendered in a primary care setting; however, definitive management for acute fracture is either rendered in the ED or at surgical specialist follow-up. It is plausible that management rendered at the acute ED encounter for DRF is not affected by insurance status. A recently published RDD study on Medicare eligibility and treatment patterns in patients with trauma showed little evidence of differences in management decisions based on insurance coverage or type.^58^ Because obtaining Medicare has been associated with a substantial reduction in health care spending and delays in care among surgical patients aged 57 to 72 years,^31^ we hypothesized that Medicare coverage would facilitate outpatient care with a surgical specialist in patients of any race and ethnicity. Yet, findings from the present study suggest that other factors play greater roles in the use of ORIF among patients of different racial and ethnic backgrounds. Second, we must acknowledge the potential for sampling bias as a reason for the findings. The study cohort included residents of Florida, Maryland, and New York only and thus was not nationally representative. However, this selection represents a range of political and social contexts, and prior studies on Medicare eligibility have found that the most substantial reductions in racial and ethnic disparities in health care outcomes occur in individuals residing in the South.^19,40^

### Limitations

This study has a few limitations. As the study used databases, we had limited insight into clinical context, fracture characteristics, radiographic findings, and patient preferences that guided operative decision-making. Although we studied a large cohort to ascertain sociodemographic patterns in management, lower rates of ORIF use in racial and ethnic minority groups could be attributed to lessened fracture severity. Other life changes at age 65 years (eg, retirement) could explain management differences. Furthermore, other unmeasured factors, such as patient educational level and health literacy as well as surgeon-level characteristics and specialty care proximity, may explain observed differences. As our study objective was to assess management differences before and after Medicare eligibility, we used HCUP all-payer databases to identify individuals who presented to the ED with DRF.^59^ Although at Michigan Medicine, nearly all patients first present to the ED, it is possible that patients initially seek care elsewhere and these individuals would not be represented in the data set. Because of data availability, we were limited to data from Florida, Maryland, and New York; thus, findings may not be generalizable to the entire US population. However, these states are well populated, are diverse, represent a range of political and social climates, and have been used to investigate socioeconomic patterns in surgical management for a variety of conditions.^21,22,60^

## Conclusions

This cohort study with RDD confirmed that ORIF was increasingly popular in adults aged 57 to 72 years, although the findings suggest reduced ORIF use in racial and ethnic minority groups. Obtaining Medicare eligibility at age 65 years did not attenuate the existing race and ethnicity–based disparities in surgical management for DRF.

## Notes

Supplement 1. **eAppendix.** Results of Sensitivity Analysis **eTable 1.** ICD-10 Diagnosis Codes for Distal Radius Fracture **eTable 2.** ICD-10 Diagnosis Codes for Polytrauma **eTable 3.** CPT and ICD-10 Procedure Codes for Management of Distal Radius Fracture **eTable 4.** Medicare Eligibility Age-Related Discontinuities in Racial and Ethnic Disparities in DRF Management (Sensitivity Analysis: Fuzzy Regression Discontinuity Design) **eTable 5.** Medicare Eligibility Age-Related Discontinuities in Racial and Ethnic Disparities in DRF Management (Sensitivity Analysis: Excluding Medicare Advantage)

Supplement 2.
Data Sharing Statement
