# Supplementary material for: Medicare Eligibility and Racial and Ethnic Disparities in Operative Fixation for Distal Radius Fracture
Source: JAMA Netw Open. 2023 Dec 28;6(12):e2349621. doi: 10.1001/jamanetworkopen.2023.49621 (PMC10755624; doi:10.1001/jamanetworkopen.2023.49621)
Supplement: Supplement 1. — eAppendix. Results of Sensitivity Analysis eTable 1. ICD-10 Diagnosis Codes for Distal Radius Fracture eTable 2. ICD-10 Diagnosis Codes for Polytrauma eTable 3. CPT and ICD-10 Procedure Codes for Management of Distal Radius Fracture eTable 4. Medicare Eligibility Age-Related Discontinuities in Racial and Ethnic Disparities in DRF Management (Sensitivity Analysis: Fuzzy Regression Discontinuity Design) eTable 5. Medicare Eligibility Age-Related Discontinuities in Racial and Ethnic Disparities in DRF Management (Sensitivity Analysis: Excluding Medicare Advantage) [file jamanetwopen-e2349621-s001.pdf]

Supplemental Online Content

Benítez TM, Ouyang Z, Khouri AN, Fahmy JN, Wang L, Chung KC. Medicare eligibility and racial and ethnic disparities in operative fixation for distal radius fracture. *JAMA Netw Open*. 2023;6(12):e2349621. doi:10.1001/jamanetworkopen.2023.49621

eAppendix. Results of Sensitivity Analysis

eTable 1. ICD-10 Diagnosis Codes for Distal Radius Fracture

eTable 2. ICD-10 Diagnosis Codes for Polytrauma

eTable 3. CPT and ICD-10 Procedure Codes for Management of Distal Radius Fracture

eTable 4. Medicare Eligibility Age-Related Discontinuities in Racial and Ethnic Disparities in DRF Management (Sensitivity Analysis: Fuzzy Regression Discontinuity Design)

eTable 5. Medicare Eligibility Age-Related Discontinuities in Racial and Ethnic Disparities in DRF Management (Sensitivity Analysis: Excluding Medicare Advantage)

This supplemental material has been provided by the authors to give readers additional information about their work.

© 2023 Benitez TM et al. *JAMA Network Open*

eAppendix. Results of Sensitivity Analysis

The results of our fuzzy regression discontinuity analysis are presented in eTable 4. In this analysis, the expected mean use of ORIF at age 65 is less for all racial and ethnic groups, particularly Black patients, as compared to the main analysis. Thus, there is greater expected White-Black and White-Hispanic disparity than reported in the main analysis. In this analysis, there was a significant, though subtle, decline in ORIF use in White patients immediately at 65 years. In this model, White patients receiving ORIF declined 2.9 percentage points (from 36.4% to 33.5%; 95% CI=-5.3, -0.5). Conversely, in the main analysis, there was a positive adjusted discontinuity in ORIF use at age 65 in White patients, although this was not statistically significant. In both models, there was a predicted decline in ORIF use at age 65 in minoritized patients, yet none of these findings achieved statistical significance. In this model, there was no significant difference in the White-minoritized group disparity in the presence of Medicare eligibility. The results of our regression discontinuity analysis excluding patients with Medicare Advantage are presented in eTable 5. The expected means and disparities at age 65 years in the absence of Medicare are the same as those observed in the main analysis. As compared to the main analysis, the adjusted discontinuity in White-Black disparity is of lesser magnitude and does not meet statistical significance (p=0.09).

© 2023 Benitez TM et al. *JAMA Network Open*

eTable 1. ICD-10 Diagnosis Codes for Distal Radius Fracture

	Description
S52.50	Unspecified fracture of the lower end of radius
S52.5	Fracture of lower end of radius
S52.50	Unspecified fracture of the lower end of radius
S52.501 A,D,G,K,P,S	Unspecified fracture of the lower end of right radius
S52.502 A,D,G,K,P,S	Unspecified fracture of the lower end of left radius
S52.509 A,D,G,K,P,S	Unspecified fracture of the lower end of unspecified radius
S52.52	Torus fracture of lower end of radius
S52.522 A,D,G,K,P,S	Torus fracture of lower end of left radius
S52.529 A,D,G,K,P,S	Torus fracture of lower end of unspecified radius
S52.53	Colles' fracture
S52.531 A,D,G,K,P,S	Colles' fracture of right radius
S52.532 A,D,G,K,P,S	Colles' fracture of left radius
S52.539 A,D,G,K,P,S	Colles' fracture of unspecified radius
S52.54	Smith's fracture
S52.541 A,D,G,K,P,S	Smith's fracture of right radius
S52.542 A,D,G,K,P,S	Smith's fracture of left radius
S52.549 A,D,G,K,P,S	Smith's fracture of unspecified radius
S52.55	Other extraarticular fracture of lower end of radius
S52.551 A,D,G,K,P,S	Other extraarticular fracture of lower end of right radius
S52.552 A,D,G,K,P,S	Other extraarticular fracture of lower end of left radius
S52.559 A,D,G,K,P,S	Other extraarticular fracture of lower end of unspecified radius
S52.56	Barton's fracture
S52.561 A,D,G,K,P,S	Barton's fracture of right radius
S52.562 A,D,G,K,P,S	Barton's fracture of left radius
S52.569 A,D,G,K,P,S	Barton's fracture of unspecified radius
S52.57	
S52.571 A,D,G,K,P,S	Other intraarticular fracture of lower end of right radius
S52.572 A,D,G,K,P,S	Other intraarticular fracture of lower end of left radius
S52.579 A,D,G,K,P,S	Other intraarticular fracture of lower end of unspecified radius
S52.59	Other fractures of lower end of radius
S52.591 A,D,G,K,P,S	Other fractures of lower end of right radius
S52.592 A,D,G,K,P,S	Other fractures of lower end of left radius
S52.599 A,D,G,K,P,S	Other fractures of lower end of unspecified radius

© 2023 Benitez TM et al. *JAMA Network Open*

eTable 2. ICD-10 Diagnosis Codes for Polytrauma

Fractures	
Face and Skull	S02.0
	S02.1
	S02.2
	S02.3
	S02.4
	S02.5
	S02.6
	S02.7
	S02.8
	S02.9
Clavicle	S42.01
	S42.02
	S42.03
Scapula	S42.1
Upper Extremity	S42.2
	S42.3
	S42.4
	S42.9
Rib	S22.X
Spine or pelvis	S32.0
	S32.1
	S32.2
	S32.3
	S32.4
	S32.5
	S32.6
	S32.8
	S32.9
Lower extremity	S82.0
	S82.1
	S82.2
	S82.3
	S82.4
	S82.5
	S82.6
	S82.8
	S82.9
**Neurologic Injuries**	
Traumatic brain injury	S02.0

© 2023 Benitez TM et al. *JAMA Network Open*

	S02.1
	S06.0
	S06.1
	S06.2
	S06.3
	S06.31
	S06.32
	S06.33
	S09.X
Epidural hemorrhage	S06.4
Subdural hemorrhage	S06.5
Subarachnoid hemorrhage	S06.6
Other specified intracranial injurie	S06.8
Unspecified intracranial injury	S06.9
**Thoracic Injury**	
Pneumothorax, unspecified	J93
Hemothorax	J94.2
**Solid Organ Injury**	
Diaphragm	S27.8
Splenic injury	S36.0
Liver injury	S36.1
Pancreatic injury	S36.2
Stomach injury	S36.3
small intestine injury	S36.4
colon injury	S36.5
rectum injury	S36.6
other intraabdominal injury	S36.8
injury of unspecified intra-abdominal organ	S36.9
kidney or other pelvic organs	S37.0
	S37.1
	S37.2
	S37.3
	S37.4
	S37.5
	S37.6
	S37.8
	S37.9

© 2023 Benitez TM et al. *JAMA Network Open*

eTable 3. CPT and ICD-10 Procedure Codes for Management of Distal Radius Fracture

**OPEN REDUCTION AND INTERNAL FIXATION (ORIF)**	
25607	Open treatment of extraarticular distal radius fracture with or without internal or external fixation
25608	Open treatment of extraarticular distal radius fracture with internal fixation of two fragments
25609	Open treatment of extraarticular distal radius fracture with internal fixation of three or more fragments
25620	Open treatment of distal radius fracture
0PSH04Z	Reposition right radius with internal fixation device, open approach
0PSH06Z	Reposition right radius with intramedullary internal fixation device, open approach
0PSJ04Z	Reposition left radius with internal fixation device, open approach
0PSJ06Z	Reposition left radius with intramedullary internal fixation device, open approach
0PSK04Z	Reposition right ulna with internal fixation device, open approach
0PSL04Z	Reposition left ulna with internal fixation device, open approach
0PSK06Z	Reposition right ulna with intramedullary internal fixation device, open approach
0PSL06Z	Reposition left ulna with intramedullary internal fixation device, open approach
**PERCUTANEOUS PINNING / FIXATION**	
25611	Percutaneous skeletal fixation of distal radius fracture
25606	Percutaneous fixation distal radius fracture or epiphyseal separation
0PSH34Z	Reposition right radius with internal fixation device, percutaneous approach
0PSJ34Z	Reposition left radius with internal fixation device, percutaneous approach
0PSH36Z	Reposition right radius with intramedullary internal fixation device, percutaneous approach
0PSJ36Z	Reposition left radius with intramedullary internal fixation device, percutaneous approach
0PSK34Z	Reposition right ulna with internal fixation device, percutaneous approach
0PSL34Z	Reposition left ulna with internal fixation device, percutaneous approach
0PSK36Z	Reposition right ulna with intramedullary internal fixation device, percutaneous approach
0PSL36Z	Reposition left ulna with intramedullary internal fixation device, percutaneous approach
**OTHER SURGICAL TREATMENT: EXTERNAL FIXATION**	
20690	Application of a uniplane unilateral external fixation system
20692	Application of a multiplane unilateral external fixation system
0PHH35Z	Insertion of external fixation device into right radius, percutaneous approach
0PHH3CZ	Insertion of ring external fixation device into right radius, percutaneous approach
0PHH3BZ	Insertion of monoplanar external fixation device into right radius, percutaneous approach
0PHH3DZ	Insertion of hybrid external fixation device into right radius, percutaneous approach

© 2023 Benitez TM et al. *JAMA Network Open*

0PHJ35Z	Insertion of external fixation device into left radius, percutaneous approach
0PHJ3CZ	Insertion of ring external fixation device into left radius, percutaneous approach
0PHJ3BZ	Insertion of monoplanar external fixation device into left radius, percutaneous approach
0PHJ3DZ	Insertion of hybrid external fixation device into left radius, percutaneous approach
0PHK35Z	Insertion of external fixation device into right ulna, percutaneous approach
0PHK3CZ	Insertion of ring external fixation device into right ulna, percutaneous approach
0PHK3BZ	Insertion of monoplanar external fixation device into right ulna, percutaneous approach
0PHK3DZ	Insertion of hybrid external fixation device into right ulna, percutaneous approach
0PHL35Z	Insertion of external fixation device into left ulna, percutaneous approach
0PHL3CZ	Insertion of ring external fixation device into left ulna, percutaneous approach
0PHL3BZ	Insertion of monoplanar external fixation device into left ulna, percutaneous approach
0PHL3DZ	Insertion of hybrid external fixation device into left ulna, percutaneous approach
**CLOSED TREATMENT**	
25600	Closed treatment of distal radius fracture, without manipulation
25605	Closed treatment of distal radius fracture, with manipulation
29065	Application of long arm (shoulder to hand) cast
29075	Application of short arm (elbow to hand) cast
29085	Application of hand and lower forearm cast
29105	Application of long arm splint
29125	Application of short arm static splint
29126	Application of short arm dynamic splint
0PSHXZZ	Reposition right radius, external approach
0PSJXZZ	Reposition left radius, external approach
0PSKXZZ	Reposition right ulna, external approach
0PSLXZZ	Reposition left ulna, external approach
2W3CX1Z	Immobilization of right lower arm using splint
2W3CX2Z	Immobilization of right lower arm using cast
2W3CX3Z	Immobilization of right lower arm using brace
2W3CXYZ	Immobilization of right lower arm using other device
2W3DX1Z	Immobilization of left lower arm using splint
2W3DX2Z	Immobilization of left lower arm using cast
2W3DX3Z	Immobilization of left lower arm using brace
2W3DX4Z	Immobilization of left lower arm using other device

© 2023 Benitez TM et al. *JAMA Network Open*

eTable 4. Medicare Eligibility Age-Related Discontinuities in Racial and Ethnic Disparities in DRF Management (Sensitivity Analysis: Fuzzy Regression Discontinuity Design)

Racial or Ethnic Group	Expected Mean[a] (%)	Adjusted Discontinuity[b] (95% CI)	Expected Disparity[c] (%)	Adjusted Discontinuity[b] in Disparity (95% CI)	p-value
**Black**	20.7	-1.1 (-7.2, 5.1)	15.7	-1.8 (-8.5, 4.8)	0.586
**Hispanic**	26.4	-2.9 (-7.7,1.9)	10	0.0 (-5.4, 5.3)	0.995
**White**	36.4	-2.9 (-5.3, -0.5)	N/A	N/A	N/A

CI, confidence interval

[a]Column presents the expected mean at age 65 years, which is based on local linear association between the age and outcome (use of ORIF vs. all other treatments). The expected mean contain the counterfactual outcome at age 65 years in the absence of treatment (e.g., the expected outcome at age 65 years without Medicare).

[b]Adjusted discontinuity estimates are in percentage points.

[c]Column presents the expected disparity at age 65 years, which is based on the local linear association between age and outcome (use of ORIF vs. all other treatments). The expected disparity subtracts the expected mean for minoritized groups from the expected mean for White populations at age 65 years.

© 2023 Benitez TM et al. *JAMA Network Open*

eTable 5. Medicare Eligibility Age-Related Discontinuities in Racial and Ethnic Disparities in DRF Management (Sensitivity Analysis: Excluding Medicare Advantage)

Racial or Ethnic Group	Expected Mean[a] (%)	Adjusted Discontinuity[b] (95% CI)	Expected Disparity[c] (%)	Adjusted Discontinuity[b] in Disparity (95% CI)	p-value
**Black**	25.1	-6.9 (-16.0, 2.3)	12.6	8.4 (-1.3, 18.1)	0.09
**Hispanic**	28.0	-1.4 (-8.4, 5.7)	9.7	2.9 (-4.9, 10.7)	0.462
**White**	37.7	1.5 (-1.8, 4.9)	N/A	N/A	N/A

CI, confidence interval

[a]Column presents the expected mean at age 65 years, which is based on local linear association between the age and outcome (use of ORIF vs. all other treatments). The expected mean contain the counterfactual outcome at age 65 years in the absence of treatment (e.g., the expected outcome at age 65 years without Medicare).

[b]Adjusted discontinuity estimates are in percentage points.

[c]Column presents the expected disparity at age 65 years, which is based on the local linear association between age and outcome (use of ORIF vs. all other treatments). The expected disparity subtracts the expected mean for minoritized groups from the expected mean for White populations at age 65 years.

© 2023 Benitez TM et al. *JAMA Network Open*
