# Supplementary material for: Medicare Eligibility and Racial and Ethnic Disparities in Operative Fixation for Distal Radius Fracture
Source: JAMA Netw Open. 2023 Dec 28;6(12):e2349621. doi: 10.1001/jamanetworkopen.2023.49621 (PMC10755624; doi:10.1001/jamanetworkopen.2023.49621)
Supplement: Supplement 2. — Data Sharing Statement [file jamanetwopen-e2349621-s002.pdf]

Data Sharing Statement

Benítez. Medicare Eligibility and Racial and Ethnic Disparities in Operative Fixation for Distal Radius Fracture. *JAMA Netw Open*. Published December 28, 2023.
doi:10.1001/jamanetworkopen.2023.49621

Data
Data available: No

Additional Information
Explanation for why data not available: Per Healthcare Cost and Utilization Project DUA, we are prohibited from sharing raw data.
